# Regulation of macrophage polarization in chronic airway inflammatory diseases: immune interactions, metabolic reprogramming, and beyond

**DOI:** 10.3389/fimmu.2026.1923683

**Published:** 2026-09-07

**Authors:** Hongyang Zhang, Cuida Meng, Dongdong Zhu

**Affiliations:** 1 Department of Otolaryngology Head and Neck Surgery, China-Japan Union Hospital of Jilin University, Changchun, China; 2 Jilin Provincial Key Laboratory of Precise Diagnosis and Treatment of Upper Airway Allergic Diseases, Changchun, China; 3 Otolaryngology Head and Neck Surgery Research Center, Changchun, China

**Keywords:** chronic airway inflammation, allergic rhinitis, chronic rhinosinusitis, asthma, macrophage polarization, immune cells, metabolic reprogramming

## Abstract

Chronic upper and lower airway inflammatory diseases (such as allergic rhinitis, chronic rhinosinusitis, and bronchial asthma) are a group of highly heterogeneous disorders primarily characterized by immune imbalance and persistent inflammation as their core features, which seriously affect patients’ quality of life. As an important component of the immune system, macrophages can undergo functional polarization in response to distinct inflammatory microenvironments, exerting divergent pro-inflammatory or anti-inflammatory effects. They also form intricate interaction networks with other immune cells to jointly regulate the progression of chronic airway inflammation. Although the regulation of macrophage plasticity is regulated by multiple aspects, metabolic reprogramming has emerged as a pivotal mechanism modulating macrophage polarization and function. This review systematically summarizes the interplay between macrophage polarization and other immune cells, focusing on the impact of distinct phenotypes and their regulatory pathways on chronic airway inflammatory diseases. Furthermore, we discuss the therapeutic potential of targeting metabolic reprogramming, providing new insights for the precise treatment of chronic upper and lower airway inflammatory diseases.

## 1 Introduction

Chronic upper and lower airway inflammatory diseases are highly prevalent chronic conditions worldwide, including allergic rhinitis (AR), chronic rhinosinusitis (CRS), and bronchial asthma. Their incidence has continued to increase in recent years. These disorders are influenced by multiple factors, including genetic predisposition, microbial infections, and allergic responses. Typical clinical manifestations include nasal congestion, sneezing, rhinorrhea, chest tightness, and dyspnea (1). Notably, many patients suffer from multiple coexisting airway diseases (2), which seriously affects quality of life and imposes a substantial burden on healthcare systems. In recent years, the concept of “one airway, one disease” has been proposed (3). It highlights the integrated nature and underscores that it has become major public health challenges requiring urgent attention.

**Figure 1:**
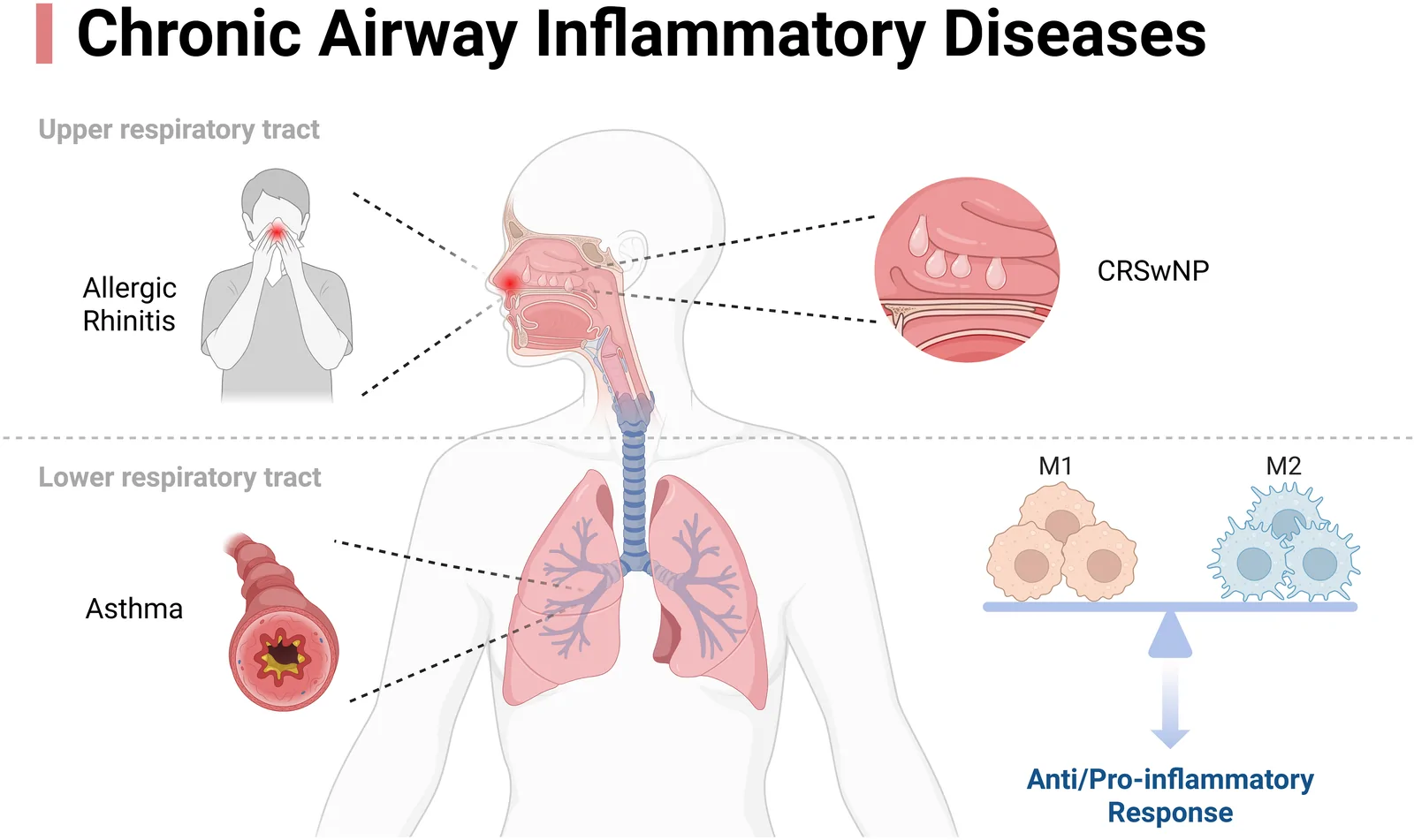
The balance of macrophage polarization regulates inflammatory responses of chronic upper and lower airway inflammatory diseases: allergic rhinitis, chronic rhinosinusitis, and asthma (created by BioRender).

Immune dysregulation is a fundamental pathogenic mechanism in these diseases. As the core cells of innate immunity, macrophages are widely distributed in human tissues (4). They present antigens to T cells as antigen-presenting cells (APCs) to initiate adaptive immune responses (5), but also regulate other immune cells by secreting a variety of cytokines and inflammatory mediators (6). It can form a complex immune network with other immune cells, jointly shape the inflammatory microenvironment, and ultimately participate in the progression or resolution of inflammation. Although macrophages can be classified into diverse phenotypes based on their activation states and functional characteristics, the classically activated M1 and alternatively activated M2 paradigms remain the most characterized and extensively investigated functional phenotypes in airway diseases. The interconversion and imbalance of these phenotypes are critical drivers of persistent airway inflammation (shown in **Figure 1**).

Notably, macrophage activation and M1/M2 phenotypic interconversion rely not only on canonical cytokines and signaling cascades but are also intimately coupled to metabolic reprogramming (7). Accumulating evidence indicates that the dynamic interplay between macrophage polarization and metabolic reprogramming participates in the course and therapeutic processes of chronic airway inflammatory diseases. For example, bone marrow-derived macrophages stimulated with the recombinant fusion protein rFlaA: Betv1, which comprises flagellin A (a Toll-like receptor 5 ligand) and the major birch-pollen allergen Betv1, adopt an activated and metabolic phenotype closely linked to M1 polarization. Upon stimulation with pathogen-associated molecular patterns (PAMPs), M1-polarized macrophages switch their metabolism to glycolysis, secrete pro-inflammatory cytokines, and boost phagocytic activity (8). Accumulating evidence suggests that metabolic reprogramming is not merely a consequence of macrophage polarization but also a central mechanism driving phenotypic transition and functional specialization (9, 10). Therefore, modulation of macrophage metabolic reprogramming to regulate polarization phenotypes and functional states may represent a potential therapeutic strategy for chronic airway inflammatory diseases.

In this review, we discuss the contribution of macrophage polarization to chronic airway inflammation, along with its interaction networks with other immune cells in chronic inflammatory diseases of the upper and lower airways. We summarize recent advances in the regulation of macrophage polarization and place special emphasis on the effects of metabolic reprogramming on macrophage phenotypes, with the goal of identifying potential therapeutic targets for chronic airway inflammatory disorders.

## 2 Macrophage polarization and metabolic characteristics

### 2.1 Macrophage polarization

In chronic airway inflammatory diseases, M1 macrophages primarily facilitate pathogen clearance during the early stage of inflammation. Their classical inducers include lipopolysaccharide (LPS) and interferon-γ (IFN-γ). M1 macrophages enhance inflammatory responses by releasing potent pro-inflammatory mediators, such as TNF-α, IL-1β, inducible nitric oxide synthase (iNOS) and reactive oxygen species (ROS) (11). Conversely, M2 macrophages are involved in Th2 responses, parasitic infections, and anti-inflammatory tissue repair (12). Under conditions of persistent chronic inflammation, they may also promote immune responses by increasing their numbers and high MHC class II expression, thereby contributing to disease progression. Thus, M2 macrophages exhibit a dual role in both pro-inflammatory and anti-inflammatory processes (13). M2 macrophages can be further subdivided into four categories. M2a macrophages are induced by IL-4 and IL-13 and participate in wound healing, fibrosis, and anti-parasitic immunity. M2b macrophages are stimulated by immunoglobulin complexes and Toll-like receptor (TLR) agonists and display dual immunomodulatory functions, including pro-inflammatory effects (secretion of TNF-α and IL-1β) and anti-inflammatory effects (secretion of IL-10). M2c macrophages are induced by IL-10, glucocorticoids, and TGF-β. They highly express scavenger receptors such as CD163 and secrete IL-10, exerting anti-inflammatory and immunosuppressive effects. M2d macrophages are activated by TLR signaling and specifically express vascular endothelial growth factor (VEGF), playing a significant role in tumor growth and immune escape (14).

Recently, research on macrophage polarization phenotypes and functions has made great progress, particularly studies on M2 populations have advanced to the refined level with the classification of M2a-d subsets. However, it should still be noted that the well-established M1/M2 classification of macrophage polarization is a nomenclature originally derived from *in vitro* cell-culture experiments, and this simplified binary paradigm cannot fully recapitulate the highly heterogeneous and plastic macrophage phenotypes observed *in vivo*, particularly within the airway microenvironment. As summarized in the consensus guidelines proposed by Murray et al. (15), macrophage activation exists along a continuous, multifunctional spectrum rather than two mutually exclusive polarized states. They also point out that the identification of equivalent subsets in humans has been less clear than in murine M1/M2 macrophages. This may be attributed to the fact that airway macrophages are further diversified by their distinct developmental origins (ontogeny). In the steady-state lung, long-lived tissue-resident alveolar macrophages are predominantly seeded from fetal-liver-derived monocyte progenitors during embryogenesis and maintained through local *in-situ* proliferation, largely independent of circulating bone-marrow-derived monocytes (16). Interstitial lung macrophage populations also have a fetal origin but can be replenished by circulating monocytes that infiltrate the lung and differentiate into macrophages (17). Furthermore, numerous studies have shown that during acute inflammation, monocytes are recruited from the bone marrow or circulation and infiltrate the lung or nasal mucosa. Distinct ontogenetic imprints shape macrophage responses to environmental stimuli, which further complicates the direct application of the classical M1/M2 framework to respiratory mucosal macrophages in asthma and chronic sinonasal inflammation.

### 2.2 Mechanisms of metabolic reprogramming in macrophage polarization

Metabolic reprogramming refers to the process by which cells reshape their metabolic programs to adapt to energy supply and functional demands under different microenvironmental stimuli, and it plays an important role in the regulation of immune cell functions (17). In macrophages, metabolic remodeling is not merely a passive consequence of energy supply, but is closely linked to their microenvironment and actively participates in polarization and functional specification (18). It was previously believed that different subtypes of macrophages had distinct metabolic characteristics, but recent studies have found that it is not a simple dualism.

Pro-inflammatory macrophages (M1 and certain M2 subtypes) typically exhibit metabolic characteristics dominated by glycolysis and the pentose phosphate pathway (PPP) (19). This shift rapidly generates ATP and provides NADPH to support ROS production and lipid synthesis. ROS act both as effector molecules involved in pathogen clearance and as signaling molecules that amplify inflammatory responses. They also interact with transcriptional regulatory networks to promote pro-inflammatory gene expression (20). Lactate, long regarded merely as a glycolytic end-product, is now recognized as a signaling and epigenetic molecule. Glutathione can enhance inflammatory signaling in macrophages through histone lactylation. Lactate induces M1 macrophage polarization via histone lactylation (e.g.H3K18la), amplifying oxidative stress and NLRP3 inflammasome activation (21).

In addition, the tricarboxylic acid (TCA) cycle in pro-inflammatory macrophages undergoes functional remodeling, with disruption at specific metabolic nodes resulting in the accumulation of intermediates such as succinate. Succinate stabilizes and activates hypoxia-inducible factor-1α (HIF-1α), thereby further reinforcing pro-inflammatory transcriptional programs within hypoxic and persistently inflamed microenvironments, ultimately maintaining and amplifying inflammatory responses (22). Simultaneously, inflammatory activation of macrophages induces the mitochondrial enzyme aconitate decarboxylase 1 (ACOD1, formerly immune-responsive gene 1, IRG1), which catalyzes the decarboxylation of cis-aconitate to produce itaconate (23). Itaconate exerts its anti-inflammatory effects through multiple complementary mechanisms: it inhibits succinate dehydrogenase (SDH), thereby remodeling the fragmented TCA cycle and limiting the accumulation of pro-inflammatory succinate (24). This metabolic feature has important pathophysiological significance in chronic airway inflammatory diseases.

In contrast, M2 subtype macrophages with anti-inflammatory and tissue repair functions tend to rely on metabolic pathways including mitochondrial oxidative phosphorylation (OXPHOS) and fatty acid oxidation (FAO), which provide sustained and stable ATP to meet long-term homeostatic and reparative demands (19). However, the role of fatty acid oxidation (FAO) in macrophage polarization has been a subject of ongoing debate. Early studies using etomoxir, a CPT1 inhibitor, concluded that FAO was required for IL-4-driven alternative (M2) macrophage activation, and proposed that oxidative metabolism supports an anti-inflammatory phenotype (25). However, this view has been challenged by genetic evidence. Nomura et al. showed that macrophages lacking CPT2, which were unable to perform β-oxidation of long-chain fatty acids, still fully polarized toward an M2 state upon IL-4 stimulation, both *in vitro* and *in vivo* (26). Subsequent work demonstrates that etomoxir inhibits M2 polarization primarily through an off-target effect: depletion of intracellular free coenzyme A, rather than through inhibition of FAO, since high concentrations of etomoxir retained their inhibitory effect even in the absence of CPT1a or CPT2 (27). Collectively, these findings indicate that long-chain FAO is largely dispensable for M2 polarization, and caution that conclusions based solely on pharmacological inhibition require re-evaluation. This controversy underscores the need for genetic models and careful dose titration when dissecting the metabolic requirements of macrophage polarization in airway disease. In conclusion, there are fascinating and complex correlations among the mutual conversion of macrophage polarization phenotypes, functions, and metabolic patterns that remain to be explored.

## 3 Key immune cells interacted with macrophages

### 3.1 Group 2 innate lymphoid cells

ILC2s are tissue-resident innate immune cells that swiftly respond to epithelial–derived alarmins (IL-33, IL-25, and thymic stromal lymphopoietin (TSLP)), subsequently producing large quantities of type 2 cytokines, including IL-5 and IL-13. Recent evidence highlights a critical cross-talk between ILC2s and macrophages. For example, during early acute rhinovirus infection, M2 macrophages secrete IL-33 to drive ILC2 expansion and mucus metaplasia (28).

**Figure 2:**
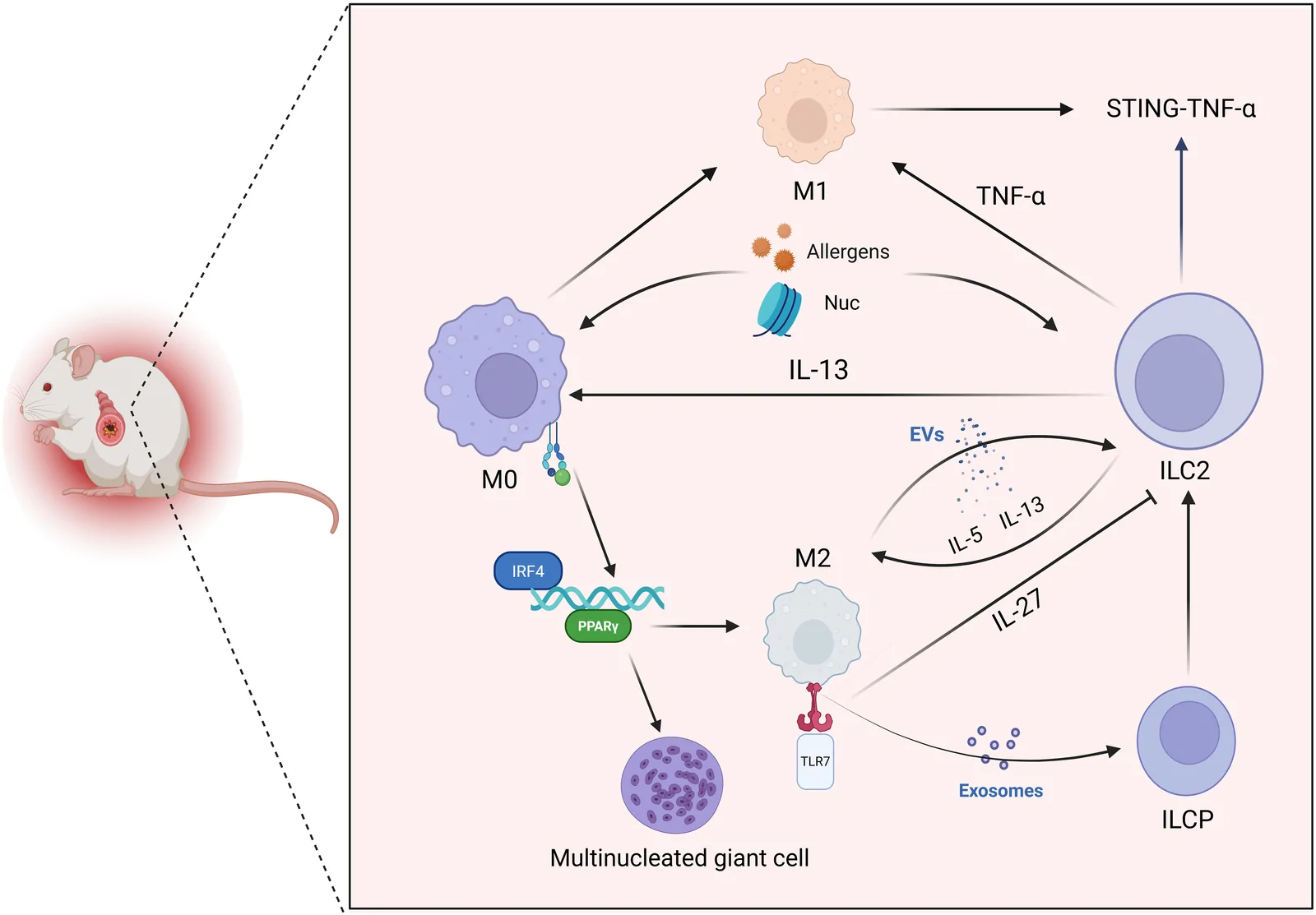
The interaction between macrophage polarization and ILC2 in chronic lower airway inflammatory diseases in mouse models. Allergens and their complexes can respectively stimulate macrophages and ILC2. Together, they enhance the pro-inflammatory M1 subtype and drive inflammation through the STING–TNF-α signaling axis; by binding to the receptor, IL-13 reprograms macrophages through IRF4, disrupts PPARγ homeostasis, transforms macrophages into pro-inflammatory M2 phenotypes, and forms multinucleated giant cells; TLR7 agonists can induce M2 pulmonary interstitial macrophages to secrete IL-27, thereby inhibiting ILC2-mediated inflammation; ILC2 internalizes extracellular vesicles derived from M2 macrophages, leading to their activation and induction of pro-inflammatory cytokines. Nuc, nucleosome; EVs, extracellular vesicles; IRF4, interferon regulatory factor 4; PPARγ, peroxisome proliferator-activated receptor gamma; TLR7, Toll-like receptor 7; ILCP, innate lymphoid cell progenitor. (created by BioRender).

Studies have shown that in a mouse model of asthma, ILC2 internalizes extracellular vesicles derived from M2 macrophages, leading to their activation and induction of pro-inflammatory cytokines (IL-5 and IL-13) (29). In HDM-induced asthmatic mice, ILC2 and M2 macrophages exert synergistic regulatory effects on asthma and allergic inflammation through arginase-1 (Arg1). IL-33 can activate ILC2 to secrete IL-13, which binds to IL-4Rα on tissue-resident alveolar macrophages (trAM). Through the IRF4 signaling axis, this interaction reprograms trAMs and disrupts their PPARγ-dependent homeostatic network. The macrophages subsequently acquire a pro-inflammatory phenotype characterized by high expression of M2 markers. These macrophages form multinucleated giant cells and recruit inflammatory cells. The interaction between ILC2 and macrophages further amplifies type 2 airway inflammation (30). Pei and colleagues report that in allergic asthmatic mice, M2 macrophages promote the differentiation of innate lymphoid cell progenitors (ILCPs) into ILC2 through secreting exosomes (31). In neutrophilic-eosinophilic inflammation in asthmatic mice, macrophages can internalize allergen-nucleosome complexes, such as Alternaria (Alt) combined with nucleosome (Nuc), thereby activating the STING pathway. Meanwhile, allergens can activate ILC2 to secrete large amounts of TNF-α, which further enhances the pro-inflammatory effects of macrophages. Together, they drive airway inflammation through the STING-TNF-α signaling axis (32). TLR7 agonists can induce M2 pulmonary interstitial macrophages to secrete IL-27, thereby inhibiting ILC2-mediated type 2 airway inflammation in non-allergic asthmatic mice (33). In AR, macrophages of patients can secrete IL-18 to promote ILC2 activation (34). **Figure 2** shows the interaction between macrophage polarization and ILC2 in the above-mentioned chronic lower airway inflammatory diseases in mouse models. ILC2 and macrophages, particularly M2 macrophages, appear to play a synergistic role in inflammatory progression. Given the close relationship between ILC2 and epithelial cells, their role in regulating macrophage polarization warrants further investigation.

### 3.2 Th2 cells

Th2 cells, which differentiate from Th0 cells upon IL-4 stimulation, are key immune cells in type 2 inflammation (35). They form complex inflammatory feedback loops with macrophages. Macrophages not only act as antigen-presenting cells (APCs) to transmit antigenic information to Th2 cells, but also regulate their activation and cytokine production (36). Th2 cells secrete IL-4, IL-5, and IL-13, thereby promoting M2 polarization of macrophages and contributing to tissue repair and the resolution of inflammation (37).

In AR, it has been found that pathogenic Th2 cells (Th2A cells) can secrete more IL-5 and IL-9 when stimulated, and are strongly associated with clinical symptoms in patients (38). In a mouse model of AR, Hu and colleagues suggest that IL-18 released by macrophages through the NLRP3 pyroptosis pathway induces T cell differentiation toward Th2 cells (39). In the same model of AR, M2 macrophages can produce histamine through interactions involving antigens and their antigen-specific Th2 cells (40).

In asthma, serum amyloid A1 (SAA1) upregulates CCL17 expression in M2 macrophages via CD36, thereby promoting Th2 cell differentiation in a model of allergic asthmatic mice (41). In asthmatic patients and sensitized mice, endoplasmic reticulum stress drives an increase in M2a macrophages and produces IL-33 to favor Th2 polarization (42). KDM5A-mediated demethylation of the IL-10 reduces Th2 cytokine production and ameliorates airway hyperresponsiveness in allergic asthmatic mice through regulation of M2 macrophages (43). Th2 cells also promote the transformation of mouse alveolar macrophages toward an M2 phenotype and multinucleated giant cells (44). Under conditions dominated by sensitized Th2 cells, a moonlighting protein, glycolytic glyceraldehyde 3-phosphate dehydrogenase (GAPDH), LGp40 may induce the conversion of allergic M2a macrophages into non-allergic M1 macrophages, suppress Th2 cell activation, and facilitate the transition of inflammatory M2a macrophages into anti-inflammatory M2c macrophages in an allergic asthma mouse model (45). The feedback circuits of Th2 cells and macrophages jointly shape the inflammatory microenvironment in chronic airway diseases.

### 3.3 Eosinophils

Peripheral or local eosinophils have become characteristic biomarkers of allergic diseases and parasitic infections, which are correlated with disease severity. Eosinophils secrete histamine, leukotrienes, IL-4, IL-5, and IL-13, thereby promoting allergic responses by enhancing vascular permeability or activating other immune cells (46). Macrophages play an important role in the recruitment of eosinophils through the secretion of various chemokines.

M2a macrophages derived from peripheral blood mononuclear cells (PBMCs) of AR patients release high levels of YKL-40, CCL17, CCL18, and CCL26, which have been shown to influence activity of Th2 cells and eosinophils (47). In ECRSwNP tissues of mice, M2 subtype macrophages have been demonstrated to recruit eosinophils through the secretion of CCL18, CCL22, CCL24, and CCL26 (48). In addition, single-cell and transcriptomics techniques have expanded the heterogeneity of human macrophages in immune interactions from a functional perspective. A study reports that ALOX15+ macrophages are the key drivers of type 2 immunity in the ECRSwNP subtype. Mechanistically, these cells promote a pro-inflammatory circuit by secreting chemokines to recruit eosinophils and other immune cells. Furthermore, the study validated that targeting ALOX15 with an inhibitor effectively alleviates type 2 immune hyperactivation, as demonstrated in both *in vitro* experiments and a humanized mouse model (49). Another transcriptomic analysis indicates that CCL13/CCL18-secreting macrophages in CRSwNP may be involved in eosinophil recruitment into the nasal epithelium (50).

In the context of lower airway inflammation, Ito and colleagues report that ALOX15-expressing eosinophils and pleural macrophages synergistically regulate eosinophil-mediated airway inflammation in asthmatic mice via 12/15-LOX expression. 12/15-LOX may be a key lipid-metabolizing enzyme that confers anti-inflammatory and tissue-protective functions on pleural macrophages to maintain tissue homeostasis (51). In a mouse model of asthma, M2a macrophages secrete high levels of IL-13 and chemokines, including CCL17, CCL18, CCL22, and CCL24, thereby promoting eosinophil infiltration into the lungs (52). Androgen receptor-deficient human M2 alveolar macrophages exhibit reduced release of the eosinophil chemokines CCL11 and CCL24 (53). Beyond pro-inflammatory effector functions, the regulatory role of eosinophils in inflammation has also attracted attention. Yoon and colleagues find that human eosinophils stimulated with TLR4 ligands produced conditioned media that, when supplemented with lipopolysaccharide (LPS) or palmitic acid, promoted the polarization of THP-1 macrophages toward M1 or M2 phenotypes, respectively. *In vivo* studies of mice have further shown that in obesity-induced inflammatory environments characterized by eosinophil reduction, although the total number of macrophages increases, the proportion of M2 macrophages decreases. These findings highlight the bidirectional interaction between eosinophils and macrophages and may provide insights into shared inflammatory mechanisms linking eosinophil-macrophage crosstalk with obesity-associated airway inflammation (54). Numerous studies have shown that eosinophils play an important role in promoting airway type 2 inflammation. Further exploration of the interaction network between macrophage polarization and eosinophils in type 2 inflammation is expected to provide new targets for eosinophilic inflammation.

### 3.4 Mast cells

Mast cells are key effector cells in inflammatory responses, particularly in type I hypersensitivity and neuroimmune interactions. Activation of FcϵRI or MRGPRX2 on the cell surface induces degranulation, leading to the release of histamine, leukotrienes, and other mediators (55). These mediators subsequently trigger vascular smooth muscle contraction and promote glandular secretion, resulting in symptoms such as nasal congestion, rhinorrhea, and dyspnea (56).

In AR patients, histamine released from mast cells acts synergistically with IL-4 via the H2 receptor (HRH2) to increase chemokine expression in macrophages (47). In the lower airway, upon human FcϵRI activation, serotonin and GM-CSF derived from mast cells promote macrophage activation and polarization toward M2 phenotype. In response to serotonin, M2 macrophages increase their production of IL-8 and TGF-β1 to promote the airway and vascular remodeling observed in asthmatic patients. Despite its pro-asthmatic influence, macrophages treated with serotonin show an increase in the expression of anti-inflammatory cytokines with a concomitant decrease in pro-inflammatory cytokines (57). Histamine released from human mast cells in highly purified lung tissue upregulates H1 receptor (HRH1) expression in M2 macrophages and induces the release of IL-6, IL-1β, and TNF-α in a concentration-dependent manner, thereby enhancing chemotaxis and altering migratory dynamics (58). *In vitro* human studies, epinephrine acts on β2 receptors expressed on bone marrow-derived M2a macrophages, shifting them toward an M2b-like phenotype. This phenotypic shift inhibits IgE-dependent inflammatory mediator release from mast cells and alleviates allergic inflammation (59). The interaction between macrophages and mast cells is more debated in animal models. In OVA-induced allergic mice, incubation of macrophages with supernatants from IgE-activated mast cells increases the expression of M1 polarization marker iNOS while downregulating the M2 marker Arg-1 (60). In contrast, Dorothea B. Holter and colleagues report that macrophages can phagocytose mast cell granules *in vitro* models. Mast cells induce transcriptomic, metabolic, and epigenetic reprogramming in macrophages through the release of multiple mediators. This generates a distinct phenotype with both classical and alternative activation features, highlighting the complexity of intercellular interactions during immune training (61). The influence of mast cells on the polarization phenotype and function of macrophages is still complex. Also, the connection between the two in neuroimmunity remains to be worthy further explored.

## 4 Regulation and function of macrophage polarization in chronic upper and lower airway inflammatory diseases

### 4.1 Allergic rhinitis

Allergic rhinitis (AR) is a type I hypersensitivity reaction induced by specific allergens such as pollen and dust mites. The main symptoms are sneezing, rhinorrhea, nasal congestion, and nasal itching (62). As antigen-presenting cells (APCs), macrophages can phagocytize and present allergens, and also secrete various cytokines or mediators, which participate in the regulation of allergic reactions and promote or improve inflammation (63).

During the pathological progression of AR, M1 macrophages primarily exert pro-inflammatory effects through the release of IL-1β, IFN-γ, and TNF-α, promoting allergen clearance while aggravating tissue injury (64). In contrast, M2 macrophages exhibit both anti-inflammatory and pro-inflammatory properties. Stimulation by IL-4 and IL-13 induces M2 macrophages to secrete IL-10, thereby facilitating anti-inflammatory responses and tissue repair. On the other hand, CD14+CD16+CD206+ M2 macrophage numbers are positively correlated with AR symptom severity in patients, and possibly augment type 2 inflammation directly by releasing YKL-40 and a distinct set of chemokines including CCL17, CCL18, and CCL26 in AR (47). The elevated levels of IL-4 and IL-13 during AR progression induce macrophage polarization toward the M2a subtype, which may represent a predominant M2 phenotype in AR. Zhang and colleagues first demonstrate that during the pollen season in patients with seasonal allergic rhinitis (SAR), the proportion of CD206+CD86- M2 macrophages (representing M2a and M2c subsets) is higher than baseline levels. At the completion of sublingual immunotherapy (SLIT), the percentage of CD206-CD86+ M2 macrophages (M2b-like regulatory macrophages) within the M2 population is significantly increased compared with baseline and peak pollen season (65).

Multiple studies have shown that M1/M2 polarization and interconversion in AR are regulated by epigenetic mechanisms and cytokine signaling. In both Human Nasal Epithelial Cells (HNEpCs) and AR mice, METTL3-mediated m6A modification promotes the nuclear export of circCDKAL1 via YTHDC1. CircCDKAL1 then interacts with IGF2BP2 and regulates the JARID2/HMGB1 axis, promoting M1 polarization. This pathway also impairs nasal epithelial barrier function and cell adhesion in AR (66). TET2 deficiency reduces m5C demethylation levels of KLF4 and ROCK1 mRNA, resulting in enhanced M2 polarization under allergic conditions in mice (67). Exosomal transfer of GAS5 promotes M1 polarization by inhibiting mTORC1/ULK1/ATG13-mediated autophagy and activating the NF-κB signaling pathway in AR mice (68). Human placental extract (HPE) inhibits NLRP3 inflammasome-mediated M1 inflammatory responses through IRGM while promoting M2-type responses, thereby exerting protective effects in AR rats (69). Mesenchymal stem cells (MSCs) significantly promote the differentiation of inflammatory M1 macrophages into anti-inflammatory M2 macrophages by secreting C-CSF, VEGF, LIF, B7-H4, and PGE2. In addition, MSCs facilitate the conversion of pro-inflammatory M1 macrophages into anti-inflammatory M2 macrophages through direct cell contact in a TSG-6 dependent manner, thereby alleviating excessive inflammation (70). However, these MSC studies are still at the animal experiments stage with no clinical trials of therapy for AR currently.

Metabolic reprogramming has emerged as a critical regulator of macrophage polarization in AR. Xu and colleagues first demonstrate that pyruvate kinase M2 (PKM2) deficiency attenuates mucosal inflammation and glycolytic activity *in vivo* models, concomitant with reduced lactate concentrations in nasal lavage fluid. Mechanistically, nuclear PKM2 dimers promote STAT3 co-activation through protein-protein interactions, thereby driving macrophage metabolic reprogramming and subsequent inflammatory responses (71). Yan et al. report that glutamine metabolism may regulate macrophage polarization in AR. Glutamine levels are elevated in the nasal mucosa of patients with AR, and glutamine supplementation promotes an M2-like macrophage phenotype in an experimental AR model. Multi-omics analyses identify FGFR1 as a potential mediator of this process, suggesting a link between glutamine metabolism and macrophage polarization in AR (72). Additionally, the tryptophan-metabolizing enzyme IL4I1 is upregulated in macrophages and nasal mucosa of AR patients; through activation of the aryl hydrocarbon receptor (AhR), IL4I1 facilitates M2 polarization, enhances epithelial IL-33 expression and STAT6 phosphorylation, and exacerbates epithelial mesenchymal transition (EMT) (73). Notably, these findings establish a mechanistic link between tryptophan metabolism and macrophage-mediated tissue remodeling in AR.

Despite these advances, the integration of macrophage polarization and metabolic reprogramming in AR pathophysiology remains incompletely understood. AhR-dependent eicosanoid signaling has been implicated in alveolar macrophages regulation (74). The nasal cavity is the primary interface for airborne pollutant exposure, and AhR is expressed in resident nasal cells (73). Whether this pathway operates in nasal mucosal macrophages remains unclear. Further studies should determine whether AhR-mediated arachidonic acid metabolism regulates macrophage reprogramming in the nasal microenvironment and contributes to AR.

### 4.2 Chronic rhinosinusitis

Chronic rhinosinusitis (CRS) is a heterogeneous disease with nasal obstruction, purulent discharge, and facial pain as the core symptoms. Its pathogenesis relates to multiple factors such as genetic susceptibility, microbial infection, and environmental exposure (75). Clinically, CRS is often classified into two main subtypes: chronic rhinosinusitis with nasal polyps (CRSwNP) and chronic rhinosinusitis without nasal polyps (CRSsNP) (76). Based on the immunopathological characteristics, chronic rhinosinusitis can be categorized into non-eosinophilic CRS (nECRS) and eosinophilic CRS (ECRS). nECRS is dominated by Th1/Th17-mediated inflammation, while ECRS is dominated by Th2 type inflammation (77). It is worth noting that in recent years, CRSwNP, especially refractory ECRSwNP under the background of type 2 inflammation, has attracted increasing attention due to the mechanisms underlying persistent inflammation and polyp recurrence (78). Therefore, the following section will mainly focus on CRSwNP.

**Figure 3:**
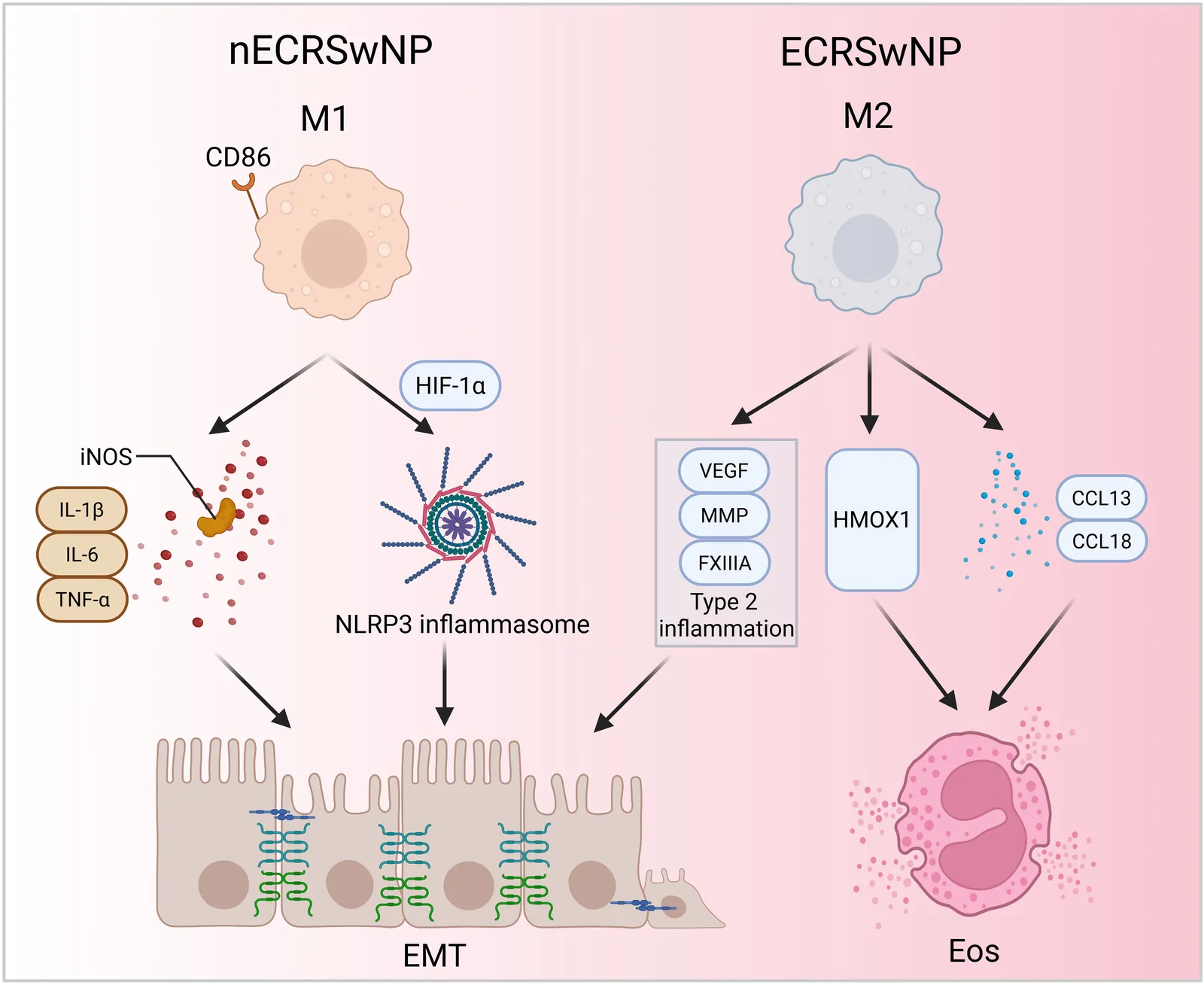
Human macrophages with different polarization play their respective roles in the course of CRSwNP of different immune subtypes. Transcriptome analysis indicates that in patients with CRS, M1 macrophages are the predominant immune cell subset in nECRSwNP. M1 macrophages produce iNOS and NLRP3, releasing pro-inflammatory cytokines that induce epithelial-mesenchymal transition. In contrast, M2 macrophages are closely associated with ECRSwNP. VEGF, MMP, and FXIIIA play essential roles in angiogenesis and edema, together mediating epithelial-mesenchymal transition. HMOX1 and CC motif ligands are correlated with infiltration of eosinophils. VEGF, vascular endothelial growth factor; MMP, matrix metalloproteinase; FXIIIA, activated coagulation factor XIII; HMOX1, heme oxygenase 1; EMT, epithelial-mesenchymal transition; Eos, eosinophil (created by BioRender).

Human macrophages with distinct polarization phenotypes exert different roles in the two inflammatory endotypes of CRSwNP (shown in **Figure 3**). Transcriptome analysis indicates that M1 macrophages and activated CD4 (+) memory T cells are the predominant immune cell subsets in nECRSwNP (79). M1 macrophages can express CD86, produce iNOS and NLRP3, releasing pro-inflammatory cytokines (IL-1β, IL-6, and TNF-α) that impair nasal epithelial cell viability and disrupt tight junction integrity, thereby inducing epithelial mesenchymal transition (EMT) and apoptosis (80).

In contrast, M2 macrophages are closely associated with ECRSwNP (79). In the context of eosinophilic inflammation, M2-derived HMOX1 is strongly correlated with eosinophil chemotactic genes and may serve as a novel diagnostic biomarker and potential regulator of inflammation in ECRSwNP (81). A spatial transcriptomic study identified TGM2+ macrophages as an eosinophilic inflammation-associated macrophage subset in nasal polyps from patients with ECRS, including those with comorbid asthma. These cells exhibited features of pro-fibrotic alternatively activated macrophages, and their abundance was strongly associated with local eosinophilic infiltration and systemic disease severity (82). Regarding polyp growth, while proinflammatory (CCL4L2 high) macrophages do not vary in numbers significantly, immunoregulatory M2-like macrophages (MRC1 and VEGFA high) are significantly elevated in CRSwNP compared with CRSsNP (50). M2 macrophages can secrete FXIIIA, VEGF, and MMP under the background of type 2 inflammation, and induce EMT and extracellular matrix remodeling by promoting fibrinogen cross-linking and angiogenesis, which eventually leads to polyp growth and recurrence (83). ALOX15(+) M2 macrophages are specifically increased in ECRSwNP patients and may be involved in epithelial remodeling by producing CCL13 (84). Co-culture of patients’ primary nasal mucosa epithelium with IL-4/IL-13 stimulated M2-like THP-1 macrophages can promote EMT by up-regulating the expression of related genes such as vimentin and TWIST1. Meanwhile, expression of MMP12 by M2 macrophages can induce EMT in nasal mucosal epithelial cells (85). Besides clinical studies, animal studies have also well proved these findings. APOE expression is elevated in M2c macrophages in both subtypes of CRSwNP patients. In murine models, it promotes fibroblast expression of MMP2 and MMP9 through the LRP1–ERK signaling pathway, thereby facilitating extracellular matrix (ECM) remodeling (86).

Multiple pathways and mediators can regulate macrophage polarization in CRSwNP. ADAM28 promotes EMT and damages tight junctions in nECRSwNP by inducing M1 polarization of macrophages. Up-regulation of SLAMF8 in THP-1 cells enhances M1 polarization, which is especially closely related to the severity of nECRSwNP (80). Air pollution-induced exosomal miRNA-19a and miRNA-614 promote the polarization of pro-inflammatory M1 macrophages by regulating the expression of RORα in the human respiratory mucosa microenvironment (87). Activation of mTOR/HIF-1α/VEGF axis promotes M1 polarization in nECRSwNP patients (88). HIF-1α also induced the expression of NLRP3 in M1 macrophages in ECRSwNP mice (89). Inhibition of the JAK/STAT3 pathway suppresses M2 polarization and remodeling of patients’ nasal mucosa. Further studies in mouse models show it delays the progression of CRSwNP (90). INPP4A deficiency promotes M2 polarization and amplifies eosinophilic inflammation in ECRSwNP mice (48). Down-regulation of small ubiquitin-like modifier (SUMO)-specific protease (SENP)3 increases M2 macrophage activation in nasal mucosal inflammation and contributes to polyp formation in mice (91). Inhibition of NOS2 and NOX1 improves the antioxidant capacity of human M2 macrophages (92). In addition, MIF, CSF1R, Tim-3, and AXL are shown to participate in the recurrence of nasal polyps in patients by promoting the polarization of M2 macrophages (93–96).

The interaction between macrophage polarization and metabolic reprogramming has garnered increasing attention in CRSwNP. Alternatively activated macrophages exhibit distinct metabolic reprogramming in ECRSwNP. Single-cell analysis of patients’ tissues demonstrates that monocyte-derived and activated tissue-resident macrophages show enhanced glycolysis, tricarboxylic acid (TCA) cycle, and oxidative phosphorylation (OXPHOS), which are closely associated with their alternative activation phenotype. These metabolic alterations support increased cell-cell interactions between macrophages and epithelial/stromal cells, thereby contributing to type 2 inflammation and tissue remodeling in ECRSwNP (97). SIRT5, as a metabolic regulator, promotes M2 polarization by enhancing glutamine catabolism, thereby contributing to ECRSwNP development in mice (98). Based on the high postoperative recurrence rate of nasal polyps in patients with metabolic syndrome, Zhou and colleagues use bioinformatics analysis and reverse transcription experiments to verify that ALOX5, HMOX1, and PLA2G7 are the core biomarkers related to macrophage polarization and metabolic reprogramming in CRSwNP patients (99). Further systematic investigation and validation of these potential metabolic biomarkers *in vivo* models and the real world may facilitate the development of novel therapeutic targets aimed at reshaping macrophage polarization through metabolic pathways, particularly for refractory eosinophilic CRSwNP.

### 4.3 Asthma

Bronchial asthma is a heterogeneous disease characterized mainly by chronic airway inflammation and airway hyperresponsiveness. Its main symptoms include recurrent wheezing, shortness of breath, and chest tightness. Pathologically, asthma manifests as airway remodeling, which is characterized by smooth muscle spasms, increased mucus secretion, and inflammatory cell infiltration (100). Macrophages involved in asthma include alveolar macrophages, interstitial macrophages, pleural macrophages, etc. (51, 101). These cells not only recruit and activate eosinophils and Th2 cells, but also secrete various cytokines and mediators that contribute to airway remodeling. In the pathological process of bronchial asthma, macrophages exhibit significant functional heterogeneity: M1 macrophages primarily exert pro-inflammatory effects. For example, leptin-induced necroptosis of M1 macrophages exacerbates HDM-induced airway inflammation in mouse asthma model (102). On the other hand, M2 macrophages exhibit dual effects of pro-inflammatory and anti-inflammatory. Arg-1, a marker of M2 macrophages, promotes the biosynthesis of polyamines and proline, which triggers bronchial smooth muscle spasms, increases mucus secretion, stimulates cell proliferation and promotes collagen deposition, thus participating in airway structural remodeling both in patients and mice (20). M2 macrophages promote airway inflammation through the JAK3-STAT5-Fra2 signaling pathway in patients with asthma and asthmatic mice (103). The deletion of SETD2 in M2 macrophages leads to Th2-type inflammation and aggravate allergic reactions both in patients and mouse model of airway allergy (104). IL-4 can change the expression profile of inflammatory cytokines by M2 macrophages and enhance the activation of lipopolysaccharides and the reactivity of M2 macrophages to lipopolysaccharides through epigenetic transcriptomics. The synergistic effect of elongation induced by interleukin-4 priming and LPS activation is present in murine-tissue-resident and human differentiating macrophages. During allergic airway inflammation, extended synergy operates in alveolar macrophages and drives enhanced LPS-induced inflammation (105). In terms of protective effects, probiotic nucleotides (LgDNA) can reduce allergic reactions by up-regulating the expression of KDM5A in M2 macrophages and increasing the expression of IL-10 (106). Furthermore, high-dimensional analysis has demonstrated novel macrophage subsets distinct from conventional subtypes. Ly6G+ Nur77+ lung macrophages represent a functionally distinct macrophage population with a dual role in asthma, acting as early sentinels that initiate allergen-driven Th2 responses while also exerting regulatory control over inflammation resolution (107). This function-oriented classification strategy may emerge as a new direction for future research on macrophage polarization subtypes, rather than simply relying on classical markers.

Regulating macrophage polarization toward the M1 subtype is generally believed to exacerbate asthma symptoms. The IL-17C/IL-17RE axis aggravates neutrophilic asthma by promoting M1 polarization in mice (108). Sphingosine-1-phosphate receptor 4 (S1PR4) deficiency aggravates the OVA/LPS-induced pulmonary inflammation in mice along with a significant up-regulation in M1 macrophage activation, which in turn secretes amounts of inflammatory cytokines associated with the inflammatory response of neutrophilic asthma (109). CXCL10 promotes M1 macrophage polarization by inhibiting SIRT3 in a mouse model, which plays an important role in the development of asthma (110). Inhibiting M1 polarization of macrophages can alleviate asthma progression. Pirfenidone inhibits M1 polarization by regulating the STAT6/p38 MAPK pathway, providing an alternative to dexamethasone in treating severe steroid-resistant asthmatic rats induced by cyp1 (111). IL-25 inhibited LPS-induced c-Rel translocation to the nucleus via STAT3-dependent signaling, ameliorating airway neutrophilia in lungs of a mouse model, at least in part, through inhibiting macrophage M1 polarization and the expression of IL-12 and IL-23 (112).

On the other hand, an increasing number of studies show that polarizing toward M2 macrophages also exacerbates asthma. Whereas, inhibiting M2 polarization of macrophages can alleviate asthma symptoms. NOTCH4 enhances IL-13-induced M2 polarization of macrophages through the AP1 and IRF4-JMJD3 axes, contributing to the increased severity of lesions in M2 inflammatory responses of asthmatic patients (113). SERPINB10 promotes M2 polarization and airway inflammation in allergic mouse models by inhibiting the degradation of IL-4Rα and upregulating the IL-4R signaling pathway (114). The M3 receptor inhibitor tiotropium bromide inhibits M2 polarization and alleviates asthma symptoms in allergic mice (115). Luteolin (LUT) effectively reduces type 2 inflammation in asthmatic mice by modulating the IL-33/ST2-GSK3β-M2 macrophage polarization (116). Deficiency of SH2-containing protein (CIS) skewed the differentiation of monocytes toward immunosuppressive M2 macrophages, promoting a strong Th2 immune response and subsequently developing into severe asthma in the mouse model (117).

Glucose and fatty acid metabolism reprogramming play a crucial regulatory role in the polarization of asthma macrophages and airway inflammation. Itaconate, as a novel anti-inflammatory and antioxidative endogenous metabolite, ameliorates while IRG1 deficiency augments allergic airway inflammation in murine models. This may be attributed to the fact that itaconate suppressed mitochondrial events in alveolar macrophages, such as NLRP3 inflammasome activation, oxidative stress, and metabolic dysfunction. The findings first provide evidence by regulating macrophage immune responses to support the increased level of itaconate in the allergic lung (118). S100A8/9-induced macrophage dysfunction and enhanced glycolysis through the TLR4/MyD88/NF-κB pathway, while the glycolysis inhibitor 3-BP can simultaneously inhibit both M1 and M2 polarization of macrophages and alleviate symptoms of asthmatic mice (119). Formaldehyde aggravates inflammation by promoting M1 polarization of macrophages and enhancing glycolysis, resulting in upregulation of lactate production and lactate dehydrogenase A (LDHA) expression via HIF-1α in alveolar macrophages of mice (120). House dust mite (HDM) induces metabolic activation of macrophages with the production of 2-hydroxyglutarate (2-HG), which is driven by the PGE2/EP2 arachidonic acid metabolism axis. This process ultimately mediates macrophage training via the FPR2–TNF–2-HG–PGE2/EP2 axis, leading to high expression of CCL17 in M2 macrophages, distinct from conventional trained immunity (121). Fatty acid binding protein 5 (FABP5) deficiency enhances IL-4-induced M2 polarization and leads to the accumulation of long-chain unsaturated fatty acids such as oleic acid. Enhanced fatty acid β-oxidation, TCA cycle activity, and OXPHOS further activate PPAR-γ signaling and increase ATP production. These metabolic changes promote M2 polarization and aggravate allergic airway inflammation (122). Dimethyl malonate inhibits mitochondrial succinate dehydrogenase and enhances mitochondrial ROS-dependent STAT6 activation. This increases the expression of M2-associated genes, including Arg1, Ym1, and Mrc1, and aggravates OVA-induced allergic asthma *in vivo* (123).

The metabolic pathways have prompted exploration of targeted therapeutic strategies in asthma. *In vitro* models, Ephedrine alleviates LPS-induced glycolysis and M1 polarization in NR8383 cells by regulating PKM2, thereby alleviating lung injury, suggesting a therapeutic role in asthma (124). Dexamethasone suppresses OVA-induced HIF-1α-glycolysis-lactate axis and subsequent protein lactylation, both in human macrophage cell line THP-1 and allergic mice (125). In allergic asthma vivo models, lactobacillus acidophilus-derived glyceraldehyde-3-phosphate dehydrogenase (GAPDH) shifts macrophage polarization from M2 to M1 phenotype by modulating glycolytic flux, thereby reversing M2-driven Th2 cell activation and attenuating airway inflammation (45). Similarly, the glycolysis inhibitor 2-deoxy-D-glucose (2-DG) reduces M2 macrophage markers through AMPK-HIF-1α pathway inhibition, alleviating allergic airway inflammation (126). Icariin, a natural flavonoid glycoside, exerts anti-inflammatory effects by regulating macrophage activation and arginine and proline metabolism via multiple targets including JUN and ALDH, effectively suppressing M1 polarization (127). Although there are already many metabolic targets that can regulate macrophage polarization in asthma compared with AR and CRS, reports on clinical trials and translations are still scarce. **Table 1** summarizes the role of metabolic reprogramming in regulating macrophage polarization in asthma.

**Table 1:** The role of metabolic reprogramming in regulating macrophage polarization in asthma.

Model	Type of research	Regulatory factors or drugs	Regulation of macrophage polarization	Biological function	Changes in metabolism	Course of diseases	Reference
Allergic asthma (OVA-induced)	*in vitro/vivo*	S100A8/9	M1↑ M2↑	Activation of TLR4/MyD88/NF-κB signaling pathway	Promoting glycolysis	Exacerbating asthma	Ji, X et al. (2024) (119)
Allergic asthma (HDM-induced)	*in vitro/vivo*	HDM	M2↑	Activation of FPR2-TNF-2-HG-PGE2/PRE2R2 axis	Promoting arachidonic acid metabolism	Exacerbating chronic type 2 airway inflammation	Lechner, A et al. (2022) (121)
Allergic asthma (OVA-induced)	*in vitro/vivo*	FABP5	M2↑	Activation of PPARγ signaling	Promoting FA β-oxidation, TCA cycle, and OXPHOS	Exacerbating asthma	Hou, Y et al. (2022) (122)
Allergic asthma (OVA-induced)	*in vitro/vivo*	DMM	M2↑	Downregulation of SDH	Promoting mitochondrial ROS	Exacerbating asthma	He, C et al. (2025) (123)
Allergic asthma (HDM-induced)	*in vitro/vivo*	LGp40	allergic M2a↓ non-allergic M1↑ anti-inflammatory M2c↑	High GAPDH activity	Regulating OXPHOS to glycolysis	Ameliorating asthma	Chen, P et al. (2022) (45)
Allergic asthma (OVA induced)	*in vitro/vivo*	2-DG	M2↓	Inhibition of AMPK-Hif-1α-dependent pathways	Inhibition of glycolysis	Ameliorating asthma	Zhao, Q et al. (2017) (126)
Allergic asthma (OVA-induced)	*in vitro/vivo*	Icariin	M1↓	Activation of Aldh2/Aldh9a1/Nos1/Nos2/Nos3	Inhibition of arginine and proline metabolism	Ameliorating asthma	Zhu, X et al. (2024) (127)
Rat alveolar macrophages (NR8383 cells)	*in vitro*	Ephedrine	M1↓	Inhibition of 2-DG/PKM2	Inhibition of glycolysis	alleviating lung injury	Xiang, Y et al. (2024) (124)

OVA, Ovalbumin; S100A8/9, S100 calcium binding protein A8/A9; HDM, House Dust Mite; FABP5, Fatty acid binding protein 5; FA, Fatty acid; TCA, Tricarboxylic acid cycle; OXPHOS, Oxidative phosphorylation; Aldh2, Aldehyde dehydrogenase 2 family member; Aldh9a1, Aldehyde dehydrogenase 9 family member A1; Nos1/2/3, Nitric oxide synthase 1/2/3; 2-DG, 2-Deoxy-D-glucose; PKM2, Pyruvate kinase M2 isoform.

While promoting anti-inflammatory M2 polarization represents a potential therapeutic strategy, emerging evidence highlights the complexity of macrophage phenotypes in asthma. Alternatively activated macrophages, particularly uncontrolled alternative activation, can itself lead to enhanced eosinophil recruitment and release of pro-remodeling mediators that can further exacerbate asthma (101). Draijer and colleagues observe that human asthma is characterized by increased IRF5+ M1 macrophages (associated with Th1 responses) and CD206+ M2 macrophages (associated with Th2 responses), concomitant with reduced IL-10+ regulatory M2-like macrophages (128). Kuo et al. also report that in asthmatic children, all types of M2 macrophages increased, but in patients with moderate and severe asthma requiring hospitalization, M1 macrophages decrease while M2c macrophages increase (129). Given the complexity of macrophage subtypes and their diverse functions in asthma, current findings underscore the necessity of precision metabolic interventions that selectively enhance anti-inflammatory M2 subsets while avoiding pathological M2 activation. This is a critical research direction for future therapeutic development in asthma and other airway inflammatory diseases.

## 5 Conclusion and future perspectives

Based on the new progress of macrophage polarization in chronic airway inflammatory diseases, this review summarizes the crosstalk between macrophages and other key immune cells; we also analyze the roles of different subtypes of macrophages and the ways to regulate macrophage polarization in diseases from the perspectives of cytokines and mediators, signaling pathways, and especially metabolic reprogramming. The traditional M1/M2 classification has been difficult to fully explain the pathological process of airway inflammation. M1 macrophages are mainly pro-inflammatory, and inhibiting their polarization can effectively reduce inflammation. The function of M2 macrophages is obviously complex. They do not simply play an anti-inflammatory role. In the context of type 2 inflammation, M2 polarization can synergistically aggravate inflammation, mediate epithelial-mesenchymal transition, collagen deposition, and eosinophil infiltration, and participate in airway tissue remodeling. Some scholars have broken through the traditional classification and divided M2 subtype macrophages into pro-inflammatory CD206+ and IL-10+ M2-like macrophages with anti-inflammatory properties. Single-cell and spatial transcriptomics technologies may become the key to accurately distinguishing between anti-inflammatory and pro-inflammatory macrophages in airway diseases from a functional perspective in the future.

Regulating macrophage polarization is a potential strategy for the treatment of chronic upper and lower airway inflammatory diseases. At present, research on macrophage polarization in chronic airway inflammatory diseases mostly focuses on the closely related upstream and downstream molecular pathways, but lacks deep research on internal metabolic regulation. The use of metabolic reprogramming to regulate macrophage polarization has attracted more attention in numerous inflammatory diseases. However, the depth of evidence remains uneven across airway diseases. Asthma has been more extensively studied than AR and CRS, potentially due to differences in disease burden, research activity, and the availability and maturity of animal models. Translating macrophage metabolic reprogramming into clinical applications also remains challenging. Metabolic pathways are widely involved in physiological processes, raising concerns about specificity, systemic effects, and safety. Disease heterogeneity further complicates clinical translation. Moreover, most current evidence comes from animal models. Human studies and well-characterized clinical cohorts remain limited. Future studies should consider macrophage heterogeneity and disease-specific metabolic contexts using human samples and clinically relevant models. Targeted delivery strategies, including nanomedicine-based approaches, may help selectively modulate macrophage metabolism while minimizing systemic effects. These approaches may ultimately facilitate more precise therapies for chronic airway inflammatory diseases.
